# Spatial drivers of COVID-19 vulnerability in Nigeria

**DOI:** 10.11604/pamj.2021.39.19.25791

**Published:** 2021-05-07

**Authors:** Mayowa Johnson Fasona, Chukwuma John Okolie, Adebayo Akeem Otitoloju

**Affiliations:** 1 Department of Geography, Faculty of Social Sciences, University of Lagos, Lagos, Nigeria,; 2 Department of Surveying and Geoinformatics, Faculty of Engineering, University of Lagos, Lagos, Nigeria,; 3 Department of Zoology, Ecotoxicology and Conservation Unit, Faculty of Science, University of Lagos, Lagos, Nigeria

**Keywords:** COVID-19, risk factors, vulnerability maps, COVID-19 prediction, geographic information system, Nigeria

## Abstract

**Introduction:**

the spread and diffusion of COVID-19 undoubtedly shows strong spatial connotations and alignment with the physical indices of civilization and globalization. Several spatial risk factors have possible influence on its dispersal trajectory. Understanding their influence is critical for mobilization, sensitization and managing non-pharmaceutical interventions at the appropriate spatial-administrative units.

**Methods:**

on 01 April 2020, we constructed a rapid spatial diagnostics and generated vulnerability map for COVID-19 infection spread at state level using 12 core spatial drivers. The risk factors used include established COVID-19 cases (as at 01 April 2020), population, proximity to the airports, inter-state road traffic, intra-state road traffic, intra city traffic, international road traffic, possible influx of elites from abroad, preponderance of high risk political elite, likelihood of religious gathering, likelihood of other social gatherings, and proximity to existing COVID-19 test centers. These were also tested as predictors of COVID-19 spread using multiple regression analysis.

**Results:**

the results show that 6 States - Lagos, Kano, Katsina, Kaduna, Oyo and Rivers - and the Federal Capital Territory have very high vulnerability, 17 states have high vulnerability and 13 states have medium vulnerability to COVID-19 transmission. Several drivers show a strong association with COVID-19 with the coefficient of correlation ranging from 0.983 - 0.995. The regression analysis indicates that between 96.6 and 99.0 percent of the total variation in the COVID-19 infections across Nigeria can be explained by the predictors.

**Conclusion:**

the spatial pattern of infection across the states are substantially consistent with the predicted pattern of vulnerability.

## Introduction

The dispersal of the new corona virus disease (COVID-19) around the world has shown some interesting patterns. Since the virus was first reported in the Wuhan megalopolis in Hubei Province of China in December 2019, it has dispersed around the world at a speed that is consistent with the speed at which humans also move from place to place, creating a trail of mortality. The geographic pattern of COVID-19 traffic suggests that COVID-19 dispersal around the globe expectedly correlates with the physical indices of civilization and globalization including: the global nighttime views of city lights, global pattern of urbanization, global internet users, and most importantly, the global airline traffic. The spread and diffusion of COVID-19 is dynamic and undoubtedly shows strong spatial connotations. For example, Sarfo and Karuppannan [1] identified an apparent link in COVID-19 infection trends and the regional level of Ghana's population distribution. Historically, disease mapping has been viewed as a relevant issue in public health, derived from an understanding of the underlying relationship between location and health [2-5]. Spatial mapping of diseases could provide insights into puzzles on disease outbreaks and connection between the location and diffusion of diseases [6]. New techniques in geography and allied fields have taken advantage of advances in geospatial technologies including geographic information system (GIS), remote sensing, global positioning system (GPS), and digital cartography for integrating geographic locations with time-dependent observations [7,8]. GIS is increasingly being employed in the analysis of spatial aspects of diseases, including the relationships between pathological factors and their environments, and management and analysis of disease information [6,9].

Several works have applied geospatial analysis and GIS for mapping disease distribution, prevalence and surveillance. These range from online disease mapping for infectious disease studies [6] to diseases prioritization from a public health and cartographic perspective [10]. Geographic information system and mapping has been severally deployed in surveillance analysis of diarrheal disease [11], filariasis [12], schistosomiasis [13,14], malaria [15-18], diabetes, asthma, and hypertension [5], Ebola [19,20], tuberculosis [21-24], and COVID-19 [25]. According to Franch-Pardo *et al*. [25], GIS and spatial analysis applications to COVID-19 include spatiotemporal analysis, health and social geography, environmental variables, data mining and web-based mapping. Geographic information system application underscores the importance of spatio-temporal elements for COVID-19 mitigation, decision making, planning and community action. Understanding the risks and vulnerabilities to diseases prevalence requires interdisciplinary thinking that considers several socio-environmental drivers including: population, transportation, existing health and social infrastructure, level of economic development and poverty profiles, social behaviour (including customs and traditions), climate, urbanisation, food security, migration, conflict, and globalisation, among others [26]. The “One World, One Health” concept [26-28] reflects the socio-environmental-disease linkage. Disease risk and vulnerability mapping are vital for geographical profiling and relationship to potential risk factors [2,5,29-31]. Africa is expected to be the most vulnerable to COVID-19 based on existing vulnerabilities including poverty and harsh socio-economic conditions and poor governance [32]. However, COVID-19 has so far proved to be a new vulnerability that has little regard for existing resilience structures [33]. Gilbert *et al*. [34] included Nigeria in the list of countries with the highest COVID-19 importation risk with a large population potentially exposed to COVID-19 infection risk. Nigeria was also listed as part of the 13 top priority countries identified by the World Health Organisation (WHO) on the basis of the volume of travel and direct links to China [34,35]. In Nigeria, the COVID-19 index case announced on 27^th^ February 2020 was a 44-year old Italian citizen who arrived the Murtala Muhammed International Airport, Lagos, on 24^th^ February 2020 aboard Turkish airline from Milan, Italy [36]. Since then, the disease has spread to other parts of the country moving from an imported and elitist pattern to community transmission [37].

Deriving from the global dispersal pattern, the transmission pathways of COVID-19 in Nigeria can be modeled from global (i.e. other continents to Africa through air and sea traffic), continental (from African countries to Nigeria through air and sea traffic), regional (from West Africa sub-region to Nigeria through air, sea, and road traffic), national (interstate movement in Nigeria through air, road, rail and water traffic), states (intra-states and inter LGAs through road, rail and inland water traffic), Local Government Areas (LGAs) and cities (intra LGAs and intra-cities/intra-towns traffic through roads, and inland water in some locations), and community or neighborhood (neighbor-to-neighbor transmission through road and person-to-person). Maritime, rail and road travels were reported as the most important pathways for the transmission of the 1918-1919 influenza pandemic that killed about 500,000 Nigerians [38]. More disease and death resulting from the pandemic was also reported in the urban areas compared to rural areas [38]. It is very likely this pattern would also hold for COVID-19. However, most of the mapping efforts by researchers in Nigeria have been directed at analysis and cartographic representation of the spatial distribution of the disease across the states. There is little evidence of research integrating social and environmental dynamics for mapping, analysis and prediction of the risks or exposure and to construct possible geographic pathways of spread of COVID-19 within Nigeria. The index case for COVID-19 in Nigeria was announced on 27 February 2020. There were 131 COVID-19 infection cases across 12 States and the Federal Capital Territory (Abuja) as at 30 March 2020 when Nigeria began a lockdown. Between 1^st^ and 5^th^ April 2020, we examined some possible geographic pathways of COVID-19 spread and constructed a rapid spatial diagnostics and generated a COVID-19 vulnerability map for Nigeria at State level. Cumulative COVID-19 infection records declared daily by the Nigeria Center for Disease Control (NCDC) are plotted on the Vulnerability map to see the pattern and level of agreement. This paper discusses the constructed vulnerability map and the extent to which the drivers explain COVID-19 infection pattern across Nigeria.

## Methods

**Study area:** Nigeria is located in West Africa with an area of about 923,769 km^2^ roughly defined by Latitudes 4° to 14° North and Longitudes 2°45´ to 14° East. It shares land borders with Benin Republic in the West, Cameroon Republic in the East, Niger Republic in the North and Chad Republic in the North East. It is also bordered by the Atlantic Ocean in the South. Nigeria is Africa´s largest economy with a population of about 201 million [39] and average density of 216.6 persons per square kilometer. The GDP per capita is about 2,320 [40]. Nigeria is also West Africa´s economic power house and major commerce and business destination. Despite the low per capita GDP, the large market attracts migrant influx from across the West Africa sub-region and from across Africa. With five major international airports, Nigeria is well opened to the rest of the World. In particular, the traffic between Nigeria and China, USA, Europe and the Middle East is very high due to heavy dependence on imports of capital and consumer goods from these countries and regions.

**Figure 1:**
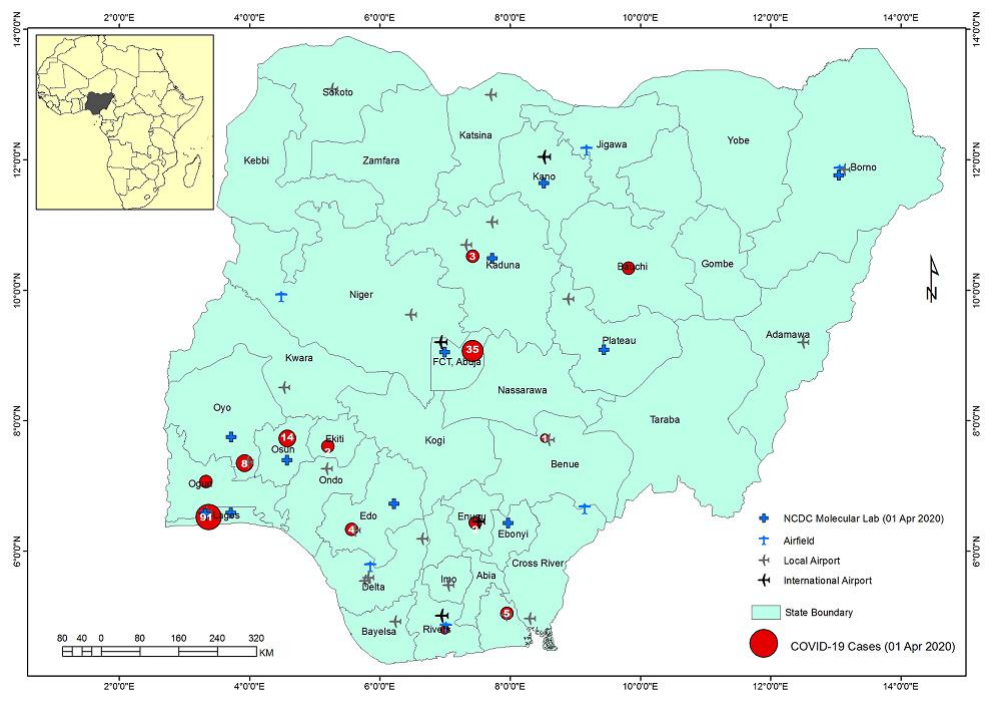
Nigeria - administrative units, airports and COVID-19 cases at 01 April 2020

**Figure 2:**
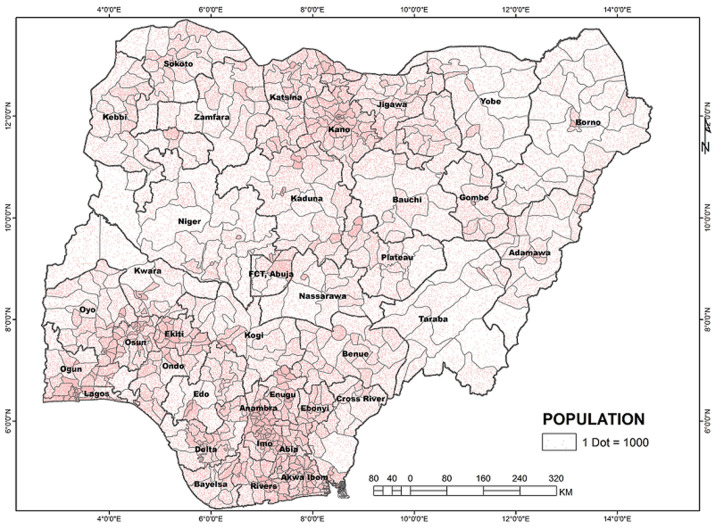
Nigeria - population density

Nigeria is divided into 36 states and a Federal Capital Territory (FCT) (Figure 1). The states are divided into Local Government Areas (LGAs) with 774 LGAs across the nation; and the LGAs divided into electoral wards. Within Nigeria, the highest population clusters are: the South-West corridor, with the epicenter around Lagos and stretching to Ekiti, Ilorin, and Lokoja axis; the Mid-West and South-Eastern areas stretching from Benin City to Enugu, Port-Harcourt and Calabar axis; the Central North with epicenter around Kano and stretching to Katsina, Zaria-Kaduna, Dutse and Hadejia axis; the less expansive areas around the Sokoto-Birni Kebbi axis; the emerging conurbation around the Abuja-Jos axis in Central Nigeria; and the state capitals (Figure 2). Internally, Nigeria is well connected mainly by air and road networks. Rail transport is currently undergoing revitalization and has not commanded the heavy traffic as it used to in the 1970s and 1980s. Commercial water transportation is limited to the interior riverine areas and riparian communities along the major rivers.

Nigeria is a rapidly urbanizing country with urban population standing at about 50.3% in 2018 [41]. Lagos is a primate city and Nigeria's only megacity with an estimated population of about 14 million in 2020 and projected to reach 21 million in 2030 [41]. Lagos is the most important economic, commercial, social and cultural center of the country, and also the major international gateway to Nigeria. With a more developed infrastructure compared to other cities in Nigeria, the traffic from Lagos to other urban areas is very high. It is not surprising that about 34% of COVID-19 cases in Nigeria have been recorded in Lagos. Every urban dweller in Nigeria has kinsmen or family in the rural area. This generates increased internal traffic between the rural and urban areas. Generally, the north-south and west-east traffic by road between the cities and urban spaces are very heavy for economic, trade, political, social, and religious reasons. This presents a formidable risk factor for COVID-19 diffusion around the country.

**COVID-19 vulnerability and spatial diffusion drivers:** vulnerability is a condition of a person or group and their situation determined by physical, social, economic, environmental, cultural and institutional conditions or processes that increase their susceptibility and influence their capacity to anticipate, cope with, resist and recover from the impact of a hazard [42-45]. Vulnerability assessment is carried out to recognize, measure, understand and predict risk as information basis for mitigation and prevention strategies [43]. Effective preparedness based on data and information has the propensity to reduce exposure and susceptibility which reduces vulnerability and overall impacts. Vulnerability embodies the hazard risk elements and their thresholds/magnitude and human capacity (or lack of capacity) to respond across space. Vulnerability assessment is critical for understanding patterns of exposure to risk, assets and resources available to respond to risk, and critical areas (hotspots) across spaces that require priority or discretional attention. It is also important for deciphering where situations are likely to result into emergencies and for prioritizing emergency response and resource allocations, including place-based and context specific interventions to reduce risk exposure and impacts, and for providing critical information to the people affected.

Generally, some key issues important to assessing vulnerability include: defining the purpose of the assessment (e.g. disease pandemic such as COVID-19, disaster management, climate change etc.), the associated drivers/indicators to be used, how to represent complexity and integrate qualitative and quantitative data, and the granularity or spatial scale at which data will be collected and analyzed (e.g. community, district, local government, or state levels). The COVID-19 pandemic belongs to the class of risk with largely uncertain probability of occurrence and well-defined maximum damage potential [46]. Its uncertain probability of occurrence leaves little time for preparedness, while the clear damage potential makes the pandemic to overwhelm even the most advanced economies with resources, systems and institutions for preparedness and response. Hence, a rapid vulnerability assessment, using available physical, social, economic, health, demographic and institutional data, to understand the spread pattern and possible diffusion trajectory may help to understand the pathways for intensifying non-pharmaceutical interventions.

While the virologists and biomedical scientists are turning to their sequencing machines to try and track the genomes of COVID-19 and understand the strains of the virus circling around the globe, it is also pertinent to look at the different possible geographic pathways of spread of COVID-19 within Nigeria. This is also important from the preventive and disaster-preparedness viewpoint. It is critical for managing mitigation and non-pharmaceutical interventions at the appropriate spatial-administrative units. Several physical, social, economic, health, and organization/governance drivers have possible influence on the transmission and trajectory of COVID-19 dispersal. However, availability of data on many of these at a fine spatial scale remains a challenge. After the index case was announced on 27^th^ February and Nigeria began a lockdown on 30^th^ March, between 1^st^ and 5^th^ April we examined some possible geographic pathways of COVID-19 spread and constructed a rapid spatial diagnostics and generated a COVID-19 vulnerability map for Nigeria at state level.

Due to time and data constraints, 12 core drivers were parameterized for this exploratory study. These are: 1. Established COVID-19 cases (as at 1^st^ April 2020); 2. Population; 3. Proximity to the airports; 4. Inter-state road traffic; 5. Intra-state road traffic; 6. Intra city and community road traffic; 7. International road traffic; 8. Possible influx of elites from abroad (imported cases); 9. Preponderance of political elite (imported cases); 10. Likelihood of religious gatherings; 11. Likelihood of other social gatherings (markets, weddings, funerals, etc.); 12. Proximity to existing COVID-19 test centers (as at 1^st^ April 2020). Some of these factors (especially on transportation) are consistent with those identified as being responsible for the diffusion of the 1918-1919 influenza pandemic [38]. Other factors are related to undermining physical (social) distancing which can exacerbate community transmission of COVID-19.

**Assumptions and parameterization:** several assumptions were made which led to the parameterization of the drivers in a GIS environment. The assumptions are as follows: 1. Established COVID-19 cases (as at 1^st^ April 2020): states with COVID-19 confirmed cases and states that share boundary with infected states are more predisposed to COVID-19 spread; 2. Population: states with higher population are more vulnerable to the spread of COVID-19 within their borders; 3. Proximity to airports: states with commercial airports (both local and international) are at higher risk of COVID-19 spread from both outside and within Nigeria; 4. Main roads: states that are traversed by major arteries (express roads and highways) have higher risk of COVID-19 spread through movement of high risk population; 5. Road traffic from outside Nigeria: states with major international gateway arteries are more predisposed to infection coming from regional countries (West Africa); 6. Abroad elite influx: imported cases by travelling elites was significant at the onset of COVID-19. Federal and state capitals and states with large urban areas are more likely to experience infection carried from travelling elite from outside the country; 7. Political elite preponderance: a significant number of early COVID-19 infections were from travelling politicians. Federal and State Capitals and states with large urban areas are likely to experience infection brought by travelling politicians; 8. Religious gathering: Nigeria is a highly religious society with many worship centers and the large number of worshippers can lead to difficulties with adhering to physical distancing guidelines. There is higher possibility of large religious gatherings in states with larger population and urban areas, 9. Other social gatherings: Nigerians have high propensity to attend social gatherings including open markets, marriages, burials/funerals, naming ceremonies, etc. in large numbers. Higher possibility of large social gatherings is likely in states with large population and urban areas; 10. Proximity to NCDC test centers (as at 1^st^ April 2020): states with NCDC COVID-19 Test Laboratories and states close to them are more likely to receive rapid attention than those far away. The spatial data including spatial-administrative units, population density, location of airports, road networks, etc. were integrated and the parameterization and analysis implemented within ArcGIS 10.3.1 as shown in Table 1. The total obtainable mark is 122. The scores obtained by the states were classified into four categories: 50 and below (low vulnerability), 51-68 (medium vulnerability), 69-85 (high vulnerability); and 86 and above (very high vulnerability).

**Table 1:** factor parameterization

S/N	Driver/factor	Code	Overall score	Unit	Parameter	Score
1	Established COVID-19 cases (as at 01 April 2020)	Sco_cases	20	Cases	Above 100	20
					60 -100	18
					40 - 59	16
					20 - 39	14
					1 - 19	12
					Share boundary with infected state= 8	8
2	Population threshold	Sco_pop	20	Number	Above 10m	20
					7.5 - 10m	18
					5 - 7.49m	16
					2.5 - 4.49m	14
					Below 2.5m	12
3	Proximity to the airport	Sco_Aipt	10		International	10
					Local	5
4	Road	Sco_Exp	5	Passes through	Expressway	5
5	Road	Sco_MJR	5	Passes through	Highway	5
6	Road	Sco_MNR	5	Passes through	Other main roads	5
7	Road traffic from outside Nigeria	TRT_INT	7	Passes through	International highway	7
8	Abroad elite	Elt_Abrd	10	Relates to state urbanization	Lagos/Abuja	10
					Above 10m	10
					7.5 - 10m	8
					5 - 7.49m	6
					2.5 - 4.49m	4
					Below 2.5m	2
9	Political elite	Pol_ELT	10	Relates to state urbanization	Lagos/Abuja	10
					Above 10m	8
					7.5 - 10m	7
					5 - 7.49m	6
					2.5 - 4.49m	5
					Below 2.5m	4
10	Religious gathering	Rel_Gath	10	Relates to state urbanization	Lagos/Abuja	10
					Above 10m	8
					7.5 - 10m	7
					5 - 7.49m	6
					2.5 - 4.49m	5
					Below 2.5m	4
11	Other social gathering	Soc_Gath	10	Relates to state urbanization	Lagos/Abuja	10
					Above 10m	10
					7.5 - 10m	8
					5 - 7.49m	6
					2.5 - 4.49m	4
					Below 2.5m	2
12	Proximity to NCDC test centers		10	Distance	Less than 50km	5
		NCDC_testc			More than 50km	10
	Total score		122			

**Validation:** the resulting database from the parameterization are updated with daily cumulative COVID-19 infection data as reported by the NCDC. The cumulative daily cases are plotted on the generated vulnerability map to examine the extent to which the pattern of infection across the states agrees with the vulnerability map. In addition, the database was uploaded from ArcGIS to IBM SPSS Statistics 20 software. Multiple regression analysis was run to determine the extent to which the parameterized drivers help to explain the pattern of recorded COVID-19 infections across the states as at 5^th^ April (when the vulnerability map was constructed), 27^th^ April (2 months after the index case), 27^th^ May (3 months after the index case), 27^th^ June (4 months after the index case) and 27^th^ July (5 months after the index case).

## Results

**Figure 3:**
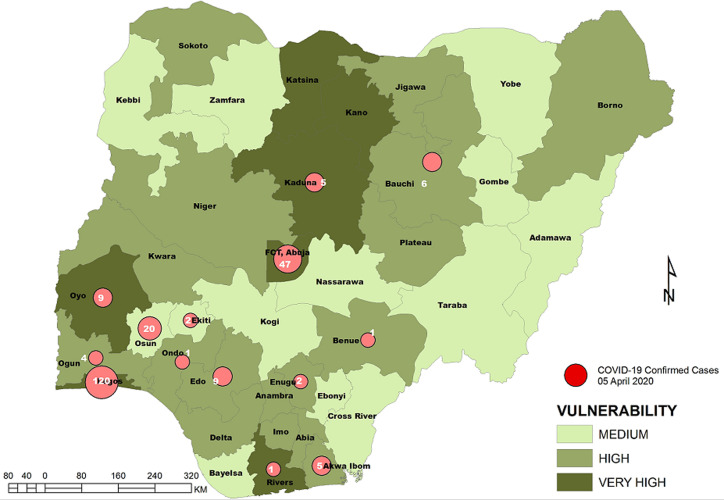
vulnerability map and COVID-19 cases, 05 April 2020

**Figure 4:**
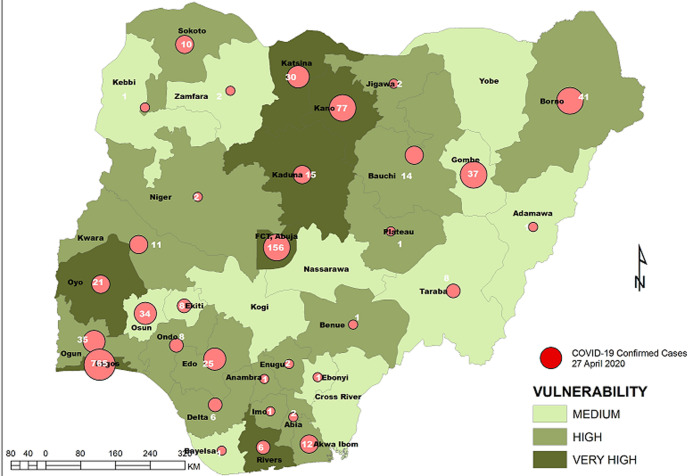
vulnerability map and COVID-19 cases, 27 April 2020

**Figure 5:**
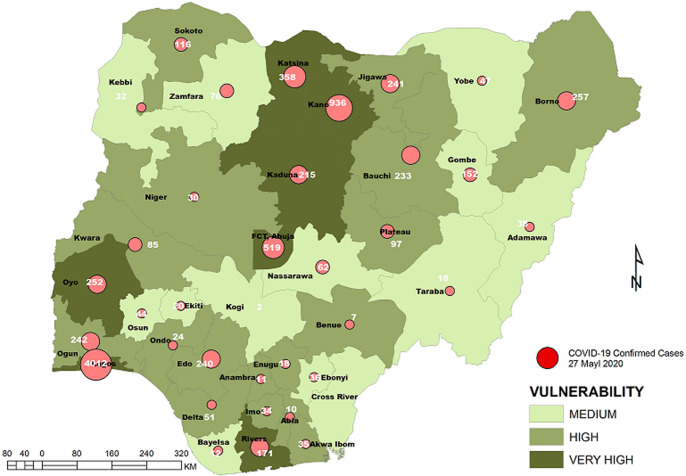
vulnerability map and COVID-19 cases, 27 May 2020

**Figure 6:**
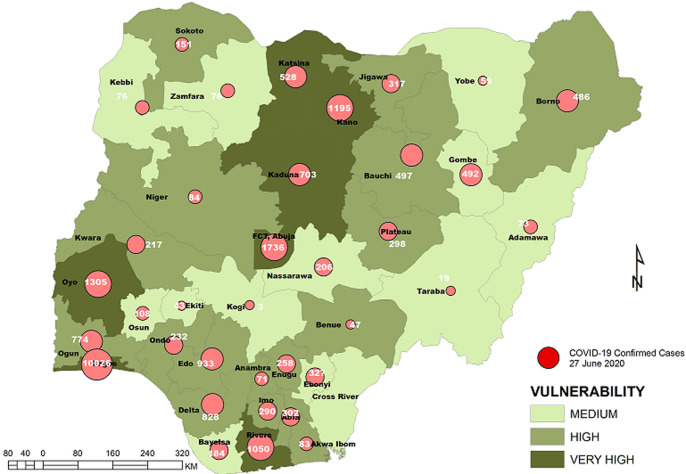
vulnerability map and COVID-19 cases, 27 June 2020

**Figure 7:**
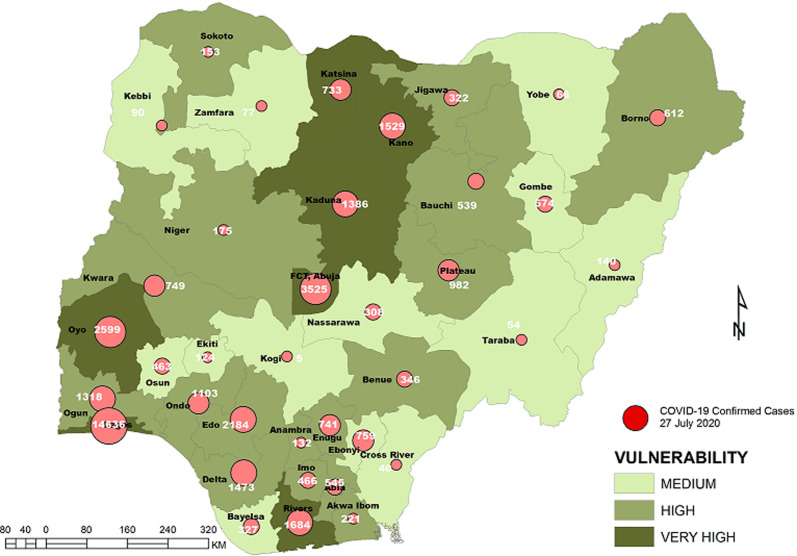
vulnerability map and COVID-19 cases, 27 July 2020

**Vulnerability maps and COVID-19 cases:** Lagos State has the highest score of 122 marks. This was followed by Kano State with 104 and FCT Abuja with 102 marks. In all, based on the 12 spatial risk drivers used in the analysis, six states - Lagos, Kano, Katsina, Kaduna, Oyo and Rivers - plus the Federal Capital Territory (FCT) have very high vulnerability to COVID-19 spread. Seventeen (17) states have high vulnerability and 13 states have medium vulnerability to COVID-19 spread in Nigeria as shown in Table 2. Figure 3, Figure 4, Figure 5, Figure 6, Figure 7 show the vulnerability maps and pattern of cumulative COVID-19 infection records across the states for 5^th^ April (when the vulnerability map was constructed), 27^th^ April (2 months after the index case), 27^th^ May (3 months after the index case), 27^th^ June (4 months after the index case) and 27^th^ July (5 months after the index case). The cumulative COVID-19 recorded infection across Nigeria increased from 232 cases (spread across 14 states and the FCT) on 5^th^ April when the vulnerability map was constructed to 1,337 cases (in 33 states and the FCT) on 27^th^ April, two months after the index case; 8,733 (in 35 states and the FCT) on 27^th^ May, three months after the index case; 24,077 (in 35 states and the FCT) on 27^th^ June, four months after the index case; and 41,180 (in all Nigeria´s 36 states and the FCT) on 27^th^ July, 5 months after the index case. The spatial pattern of infection across the states are substantially consistent with the predicted pattern of vulnerability with Lagos, FCT and Kano leading in the number of infection cases in the first two months after the index case. However, with community transmission taking over, states around Lagos including Oyo, Ogun, Ondo, as well as Edo, Delta, Rivers in the southern region have recorded strong increase in number of cases. Kaduna, Plateau and Katsina also recorded a significantly high increase. Incidentally, all these states were categorized as either very high or high in the vulnerability map. A few outliers were also observed including Ebonyi and Gombe states which recorded a strong increase in the number of cases contrary to expectations. States like Sokoto and Niger in the Northwestern axis as well as Anambra and Imo in the eastern axis did not register as much COVID-19 infections as expected.

**Table 2:** vulnerability at state level

SN	State	Score_cases	Score_Pop	Score_Airpt	Scor_Exp	Sco_MJR	Scor_MNR	TRF_INT	Elt_Abrd	Pol_ELT	Rel_Gath	Soc_Gath	NCDC_testc	Total score	Vulnerability
1	Lagos	20	20	10	5	5	5	7	10	10	10	10	10	122	Very high
2	Kano	8	20	10	5	5	5	7	10	8	8	8	10	104	Very high
3	FCT, Abuja	16	14	10	5	0	5	7	10	10	10	10	5	102	Very high
4	Katsina	8	18	5	5	5	5	7	8	7	7	7	10	92	Very high
5	Kaduna	12	18	5	5	5	5	7	8	7	7	7	5	91	Very high
6	Oyo	12	18	5	5	5	5	7	8	7	7	7	5	91	Very high
7	Rivers	12	16	10	5	0	5	7	6	6	6	6	10	89	Very high
8	Imo	8	16	5	5	5	5	7	6	6	6	6	10	85	High
9	Bauchi	12	16	0	5	5	5	7	6	6	6	6	10	84	High
10	Delta	8	16	5	5	5	5	7	6	6	6	6	5	80	High
11	Jigawa	8	16	0	5	5	5	7	6	6	6	6	10	80	High
12	Niger	8	16	5	5	5	5	7	6	6	6	6	5	80	High
13	Benue	12	16	5	5	0	5	7	6	6	6	6	5	79	High
14	Ogun	12	16	0	5	5	5	7	6	6	6	6	5	79	High
15	Plateau	8	14	5	5	5	5	7	4	5	5	5	10	78	High
16	Edo	12	14	5	5	5	5	7	4	5	5	5	5	77	High
17	Enugu	12	14	10	5	5	0	7	4	5	5	5	5	77	High
18	Ondo	12	14	5	5	5	5	7	4	5	5	5	5	77	High
19	Anambra	8	16	0	5	5	0	7	6	6	6	6	10	75	High
20	Abia	8	14	0	5	5	5	7	4	5	5	5	10	73	High
21	Kwara	8	14	5	5	0	5	7	4	5	5	5	10	73	High
22	Akwa Ibom	12	16	5	0	0	5	0	6	6	6	6	10	72	High
23	Borno	0	16	5	5	0	5	7	6	6	6	6	10	72	High
24	Sokoto	0	14	5	5	5	5	7	4	5	5	5	10	70	High
25	Kogi	8	14	0	5	5	5	7	4	5	5	5	5	68	Medium
26	Yobe	8	14	0	5	0	5	7	4	5	5	5	10	68	Medium
27	Kebbi	0	14	0	5	5	5	7	4	5	5	5	10	65	Medium
28	Osun	14	14	0	0	5	5	0	4	5	5	5	5	62	Medium
29	Cross River	8	14	5	0	5	5	0	4	5	5	5	5	61	Medium
30	Gombe	8	14	0	0	5	5	0	4	5	5	5	10	61	Medium
31	Nassarawa	8	12	0	5	0	5	7	2	4	4	4	10	61	Medium
32	Taraba	8	14	0	0	5	5	0	4	5	5	5	10	61	Medium
33	Zamfara	8	14	0	0	5	5	0	4	5	5	5	10	61	Medium
34	Ekiti	12	14	0	0	0	5	0	4	5	5	5	5	55	Medium
35	Bayelsa	8	12	5	0	0	5	0	2	4	4	4	10	54	Medium
36	Adamawa	0	14	5	0	0	5	0	4	5	5	5	10	53	Medium
37	Ebonyi	8	14	0	0	5	0	0	4	5	5	5	5	51	Medium

**Performance of risk factors:** in order to estimate the extent to which the selected drivers captured COVID-19 infection at state level across Nigeria, multiple regression analysis was run with the 12 predictors. However, three variables: scor_Exp (expressway traffic), Pol_ELT (political elite) and Rel_Gath (religious gathering) were automatically excluded for multicollinearity. Table 3 shows the multiple regression analysis of the selected predictors with recorded cumulative COVID-19 infection cases as at 5^th^ April, 27^th^ April, 27^th^ May, 27^th^ June and 27^th^ July. For all the dependent variables (i.e. cumulative COVID-19 cases for the dates shown), the R values range from 0.983 to 0.995 which indicate a good level of prediction. The coefficient of determination (R^2^) values suggest that between 96.6 and 99.0 percent of the total variation in the COVID-19 infections across Nigeria can be explained by the predictors. The ANOVA outputs also suggest that these variables have statistically significant effects on the recorded COVID-19 cases across Nigeria.

**Table 3:** multiple regression analysis of selected risk factors and COVID-19 cases

Dependent variable	Model	R	R^2^	Adjusted R^2^	Std. error of the estimate	ANOVA
COV_05 Apr	1	0.989	0.978	0.970	3.609	F(9, 27) =131.998, p <0.000
COV_27 Apr	1	0.995	0.990	0.986	14.777	F(9, 27) =289.907, p <0.000
COV_27 May	1	0.986	0.973	0.964	125.717	F(9, 27) =108.223, p <0.000
COV_27 Jun	1	0.989	0.977	0.970	284.802	F(9, 27) =129.255, p <0.000
COV_27 Jul	1	0.983	0.966	0.955	510.804	F(9, 27) =86.454, p <0.000

Predictors: (constant), NCDC_testC, TRF_INT, Sco_MNR, Sco_MJR, Soc_Gath, Scor_Airpt, Sco_Cases, Sco_Pop, Elt_Abrd

## Discussion

When the Federal Government of Nigeria implemented the lockdown on 30^th^ March 2020 as part of the measures to curtail the spread of the COVID-19, international airports were closed to eliminate imported cases from global, continental and regional transmissions. But in addition to considerations for special flights, the land borders remained open for Nigerians from other West African countries to enter the country. For example, the early COVID-19 cases in Osun State were imported by returnees from Abidjan, Cote D´Ivoire. The local airports were also closed to cut off interstate dispersal through air traffic. Perhaps, more significantly, total lockdown was imposed on Lagos (the epicenter of COVID-19 cases), and the nearby Ogun State (which shares part of the Lagos conurbation) and the FCT, Abuja. Nigeria entered the phased lockdown on 30^th^ March 2020 with 131 cases spread across 12 states and the FCT and started gradual lifting of total lockdown on Lagos, Ogun and Abuja after 35 days on 4^th^ May 2020 with 2,802 cases spread across 34 states and FCT, Abuja. Even after the ease of lockdown, the restrictions on mass gathering in social and religious places were maintained. These were done to reduce the risk of transmission to other states by road travelers from these high risk areas and prevent community transmission. There is no doubt that the lockdown constrained the spread of COVID-19 infections to an extent. But there were also newspaper reports suggesting that even during the lock down, illegal commercial transportation along the major road arteries thrived.

There is little evidence to show that spatial data and analytical results and outputs from GIS analysis were employed by the governments at any level in Nigeria to aid COVID-19 preparedness and responses. This presents a big setback to COVID-19 responses and mobilization as spatial characterization and risk of places to COVID-19 diffusion at different levels - state, LGA, town/community and neighborhood - were and still largely unknown. The lack of knowledge about COVID-19 risk characterization across states possibly informed the extreme top-down approach to mitigation of COVID-19 responses employed by the government and the very low level of awareness and lack of information and initiative for responsibility and ownership at the LGA and community levels in Nigeria.

## Conclusion

This study constructed rapid spatial diagnostics and generated vulnerability maps for COVID-19 infection spread at state level in Nigeria using 12 core spatial drivers that include established COVID-19 cases (as at 1^st^ April 2020), population, proximity to the airports, inter-state road traffic, intra-state road traffic, intra city traffic, international road traffic, possible influx of elites from abroad, preponderance of high risk political elite, likelihood of religious gathering, likelihood of other social gatherings and proximity to existing COVID-19 test centers. These were also tested as predictors of COVID-19 spread. The spatial pattern of infection across the states are substantially consistent with the predicted pattern of vulnerability. Several drivers show a strong association with the coefficient of correlation (R) values ranging from 0.983 - 0.995, and R^2^ values indicating that 96.6 - 99.0 percent of the total variation in the COVID-19 infections across Nigeria can be explained by the predictors. These results tend to support the general belief that lockdown measures limiting physical interaction are an effective decision against COVID-19. However, lockdown alone without mobilization and sensitization at the different spatial units including state, LGA, towns/villages and neighborhoods will not likely be successful in reducing community transmission. The latter requires robust understanding of the spatial risk and transmission factors and the vulnerability to COVID-19 at LGA and community levels. A robust understanding of the intrinsic differences in actual and expected/predicted community transmission across spatial units will help in resource mobilization, surveillance (including where increased testing is required), risk communication, and use of established community channels for contact tracing and dissemination of information on non-pharmaceutical interventions. These place-based and context-specific measures are critical for successful management of COVID-19 spread in many resource-limited countries around the world.

### What is known about this topic

- COVID-19 has so far proved to be a new vulnerability that has little regard for existing resilience structures;
- Understanding the risks and vulnerabilities to COVID-19 prevalence requires interdisciplinary thinking that considers several socio-environmental drivers.

### What this study adds

- Spatial drivers including population, transportation, high risk political elite concentration, and likelihood of religious and social gatherings are good predictors of COVID-19 spread in Nigeria with R ranging from 0.983 - 0.995. The R^2^ values indicates that between 96.6 and 99.0 percent of the total variation in the COVID-19 infections pattern across Nigeria can be explained by the predictors;
- A robust understanding of the intrinsic differences in spatial risk and its impact on community transmission across spatial units is indispensable for resource mobilization, surveillance, risk communication, and use of established community channels for contact tracing and dissemination of information on non-pharmaceutical interventions;
- The poor knowledge about COVID-19 risk characterization across spaces possibly informed the extreme top-down approach to mitigation employed by the government and the very low level of awareness and lack of information and initiative for responsibility and ownership by the population at LGA and community level.
